# Clinical Impact of Fluoroquinolone-Resistant *Escherichia coli* in the Fecal Flora of Hematological Patients with Neutropenia and Levofloxacin Prophylaxis

**DOI:** 10.1371/journal.pone.0085210

**Published:** 2014-01-22

**Authors:** Yong Chong, Shinji Shimoda, Hiroko Yakushiji, Yoshikiyo Ito, Takatoshi Aoki, Toshihiro Miyamoto, Tomohiko Kamimura, Nobuyuki Shimono, Koichi Akashi

**Affiliations:** 1 Medicine and Biosystemic Science, Kyushu University Graduate School of Medical Sciences, Fukuoka, Japan; 2 Department of Clinical Laboratory, Hara-Sanshin Hospital, Fukuoka, Japan; 3 Department of Blood and Marrow Transplantation, Hara-Sanshin Hospital, Fukuoka, Japan; 4 Center for the Study of Global Infection, Kyushu University Hospital, Fukuoka, Japan; University of Birmingham, United Kingdom

## Abstract

**Background:**

Fluoroquinolone prophylaxis in patients with neutropenia and hematological malignancies is said to be effective on febrile netropenia (FN)-related infection and mortality; however, the emergence of antibiotic resistance has become a concern. Ciprofloxacin and levofloxacin prophylaxis are most commonly recommended. A significant increase in the rate of quinolone-resistant *Escherichia coli* in fecal flora has been reported following ciprofloxacin prophylaxis. The acquisition of quinolone-resistant *E. coli* after levofloxacin use has not been evaluated.

**Methods:**

We prospectively examined the incidence of quinolone-resistant *E. coli* isolates recovered from stool cultures before and after levofloxacin prophylaxis in patients with neutropenia from August 2011 to May 2013. Some patients received chemotherapy multiple times.

**Results:**

In this trial, 68 patients were registered. Levofloxacin-resistant *E. coli* isolates were detected from 11 and 13 of all patients before and after the prophylaxis, respectively. However, this was not statistically significant (*P = *0.65). Multiple prophylaxis for sequential chemotherapy did not induce additional quinolone resistance among *E. coli* isolates. Interestingly, quinolone-resistant *E. coli*, most of which were extended-spectrum β-lactamase (ESBL) producers, were already detected in approximately 20% of all patients before the initiation of prophylaxis. FN-related bacteremia developed in 2 patients, accompanied by a good prognosis.

**Conclusions:**

Levofloxacin prophylaxis for neutropenia did not result in a significant acquisition of quinolone-resistant *E. coli*. However, we detected previous colonization of quinolone-resistant *E. coli* before prophylaxis, which possibly reflects the spread of ESBL. The epidemic spread of resistant *E. coli* as a local factor may influence strategies toward the use of quinolone prophylaxis.

## Introduction

Febrile neutropenia is a serious adverse event in patients with hematological malignancies, and is a common side effect of chemotherapy [1]. The presence of bacteremia related to febrile neutropenia (FN) often increases infection-related morbidity and mortality. The use of antibiotic prophylaxis, particularly fluoroquinolones (quinolones), has been known to positively affect the prevalence of febrile episodes, bacteremia, and even infection-related mortality [2]–[4]. However, the emergence of bacterial resistance to antibiotics, especially quinolones, has become a concern, and routine prophylactic use remains controversial [5]–[6]. Particularly, the prevalence of a breakthrough infection of quinolone-resistant gram-negative bacteremia, predominantly related to *Escherichia coli*, has been reported in neutropenic patients receiving quinolone prophylaxis [2], [7]–[12]. Bacteremia is presumed to develop from these quinolone-resistant *E. coli* strains after gut colonization, which is the result of antibiotic prophylaxis. In fact, quinolone-resistant *E. coli* were significantly detected in the fecal flora after ciprofloxacin or norfloxacin prophylaxis [13], [14]. According to the guidelines of the Infectious Diseases Society of America, ciprofloxacin and levofloxacin, both quinolone, are recommended candidates for antibiotic prophylaxis; however, levofloxacin is the most preferred quinolone because of its activity against gram-positive bacteria [15]. Despite these previous efforts, the effect of quinolone-resistant *E. coli* present in the fecal flora on patients receiving levofloxacin prophylaxis for neutropenia has not been examined. According to the findings of previous studies, we assumed that a significant acquisition of levofloxacin-resistant *E. coli* would be present in the fecal flora after prophylaxis in patients with neutropenia. In this study, we prospectively compared the detection rates of levofloxacin-resistant *E. coli* isolates recovered from stool cultures before and after quinolone prophylaxis for hematological patients with neutropenia.

## Methods

### Patients and Ethics Statement

From August 2011 to May 2013, 68 patients were recruited from a single hematological unit with 37 beds at Hara-Sanshin Hospital. The protocol was approved, through the ethics review process, by the Institutional Review Board of the Hara-Sanshin Hospital. Written informed consent was obtained from all registered patients before the study protocol was implemented, in order to publish these case details. Infection control measures including hand-washing promotion and isolation procedures were maintained throughout the study.

### Enrollment

Inpatients with neutropenia were enrolled in the study. Neutropenia was defined as an absolute neutrophil count of <1,000 cells/mm^3^ or a neutrophil count with a predicted decrease to <1,000 cells/mm^3^ during the following 48 h. Patients were excluded if they reported a history of antibiotic use within 90 days of the baseline measurement, were treated for allogeneic hematopoietic stem cell transplantation, presented with evidence of hepatic and/or renal dysfunctions (defined as a serum transaminase level of more than 3 times the upper limit of the normal range or as a serum creatinine level of more than 1.5 times the upper limit of the normal range), or had a history of hypersensitivity to fluoroquinolones. Patients treated for allogeneic hematopoietic stem cell transplantation were excluded from the trial because a confirmative diagnosis of febrile neutropenia is often difficult owing to the presence of other causative factors such as graft-versus-host disease and engraftment syndrome. Antimycotic agents were administered for most of the registered neutropenic patients.

### Treatment protocol

Antibiotic prophylaxis with levofloxacin at a dosage of 500 mg/day was administered to all patients for the duration of the study. Levofloxacin was administered to patients without febrile neutropenia until their neutrophil counts recovered. However, levofloxacin prophylactic treatment was discontinued when the empirical antibiotic therapy for febrile neutropenia was initiated. Febrile neutropenia was defined as: (1) fever, a single axillary temperature of >38.0°C or an axillary temperature >37.5°C lasting 1 hour, and (2) neutropenia, defined according to the aforementioned guidelines.

### Microbiology

For each patient, 2 stool samples were examined. The first sample was collected before levofloxacin administration, and the second sample after prophylaxis was discontinued. If a fever at the level suggesting febrile neutropenia was found, blood samples were also collected. If multiple organisms were detected from a single sample, they were counted and analyzed as independent isolates. An automated blood culture system (BACTEC) was used for each test. Stool samples were cultured, chiefly using 5% Sheep Blood Agar medium (BD) and CHROMagar Candida medium (BD). The species were identified using the Vitek system (bioMerieux Japan Ltd., Tokyo, Japan). Antibiotic susceptibilities were determined by the breakpoints standardized by the Clinical and Laboratory Standards Institute (CLSI; formerly the NCCLS) [16]. The screening and confirmation tests for ESBL and metallo-β-lactamase were conducted according to the recommendation of the CLSI [16]. In addition, β-lactamase producers were confirmed using a Cica β test I/MBL kit (Kanto Chemical Co. Ltd., Tokyo, Japan). *Clostridium difficile* toxin A and B were examined in stool samples using a TOX A/B QUIK CHEK kit (Nissui Pharmaceutical Co. Ltd., Tokyo, Japan).

### Study Outcomes

The primary outcome was the rates of levofloxacin-resistant *E. coli* detected in the fecal flora before and after quinolone prophylaxis for all patients. We also analyzed the association between multiple prophylaxis and the additional acquisition of levofloxacin-resistant *E. coli.*

### Statistical analysis

We powered the trial on the basis of the secondary outcome, the rates of levofloxacin-resistant *E. coli* detected in the fecal flora before and after prophylaxis for patients with the second registration for cycle 2 of chemotherapy. Previous studies investigating the use of fluoroquinolones (ciprofloxacin and norfloxacin) for the prophylaxis of febrile neutropenia showed that the rate of the newly acquired quinolone-resistant *E. coli* in the fecal flora was approximately 30% [13], [14]. Sample size calculations indicated that enrollment of at least 21 patients was required to achieve 80% power for the detection of at least a 30% acquisition rate, with a 2-sided alpha of 0.05. Data were analyzed after the study period ended. The recruitment of study participants stopped once the target sample size was achieved. Outcomes were assessed using chi-square tests. A *P*<0.05 was considered to be statistically significant. All statistical calculations were performed using SAS software (SAS Institute, Inc., Cary, NC, USA).

## Results

### Characteristics of patients registered for levofloxacin prophylaxis

The characteristics of all 68 patients enrolled in this study were shown in Table 1. Antibiotic prophylaxis was conducted for chemotherapy-induced neutropenia in almost all cases. A portion of patients were registered multiple times for sequential chemotherapy. Thirty three of all the 68 enrolls were applied for more than cycle 2 of chemotherapy. Precisely, within the first 35 enrolls, 21 patients were registered for cycle 2 of sequential chemotherapy. Within the second 21 enrolls, 12 patients were registered for more than cycle 3 of chemotherapy (Table 1).

**Table 1:** Characteristics of patients registered for levofloxacin prophylaxis.

Characteristic	Value for group
No. of patients	68
Age, mean years±SD (range)	68.0±6.4 (53–91)
Male sex	33 (48.5)
Malignant disease	
Leukemia	32 (47.0)
Lymphoma	24 (35.3)
MDS	3 (4.4)
Multiple myeloma	8 (11.8)
Other	1 (1.5)
Therapy for hematological disorders	
Chemotherapy	67 (98.5)
Autologous HSCT	2 (2.9)
No. of cycles	
cycle 1	35
cycle 2	21
cycle >3	12
mean days of prophylaxis, mean days±SD (range)	12.2±14.8 (4–96)
episodes of bacteremia	
gram-negative	1 (1.5)
gram-positive	1 (1.5)
prognosis: death	
Total	1 (1.5)
Infection-related death	0 (0.0)

Cycle 1, 2, and >3 indicate first, second, and more than third registration, respectively.

Episodes of bacteremia indicates febrile neutropenia-related bacteremia during levofloxacin prophylaxis.

Prognosis indicates death during prophylaxis and within 7 days after prophylaxis.

MDS, myelodysplastic syndromes; HSCT, hematopoietic stem cell transplantation.

### Fluoroquinolone-resistant *E. coli* in the fecal flora of patients with levofloxacin prophylaxis

The etiology of bacterial isolates in the fecal flora was compared before and after prophylaxis. The incidence of gram-negative isolates decreased from 92 of 254 (36.2%) to 17of 169 (10.1%) before and after prophylaxis, respectively. In contrast, the detection rate of gram-positive isolates significantly increased (63.8%, n = 162 vs. 89.9%, n = 152) before and after prophylaxis. The prevalence of levofloxacin-resistant *E. coli* isolates recovered from stool cultures both before and after antibiotic prophylaxis are shown in Table 2. Quinolone-resistant *E. coli* strains were detected in 11 of the 68 total samples collected before antibiotic prophylaxis. After prophylaxis, 13 of the 68 samples recovered the quinolone-resistant *E. coli*,. The rates of levofloxacin-resistant *E. coli* stool isolates before and after prophylaxis were not significantly different (*P = *0.65). ESBL producers were detected in 7 of 11 and 7 of 13 *E. coli* isolates resistant to levofloxacin before and after prophylaxis, respectively. Thus, none of ESBL-producing *E. coli* was newly acquired in the fecal flora after quinolone prophylaxis. Newly acquired quinolone-resistant *E. coli* were detected in 2 cases during the first prophylaxis (Table 2). There were no newly detected quinolone-resistant *E. coli* for patients who underwent more than 2 cycles of chemotherapy. Among other gram-negative bacteria besides *E. coli*, levofloxacin resistance was detected in 1 sample before prophylaxis and 2 samples after.

**Table 2:** Fluoroquinolone-resistance of *E. coli* isolates in fecal sample before and after levofloxacin prophylaxis.

	Before prophylaxis		After	
Sample	No. (%) of samples with quinolone-resistant *E. coli*	No. (%) of samples with ESBL-producing *E. coli*	No. (%) of samples with quinolone-resistant *E. coli*	No. (%) of samples with ESBL-producing *E. coli*
Total, n = 68	11 (16.1)^a^	7 (10.3)	13 (19.1)^a^	7 (10.3)
Each cycle				
cycle 1, n = 35	5 (14.3)	2 (5.7)	7 (20.0)	2 (5.7)
cycle 2, n = 21	4 (19.0)	3 (14.3)	4 (19.0)	3 (14.3)
cycle >3, n = 12	2 (16.7)	2 (16.7)	2 (16.7)	2 (16.7)

a *P*-value shows statistical comparison for each variable.

a *p = *0.65.

Cycle 1, 2, and >3 indicate first, second, and more than third registration, respectively.

ESBL, extended-spectrum β-lactamase.

### Mortality

In this study, febrile neutropenia-related bacteremia isolates including levofloxacin-resistant *E. coli* and *Staphylococcus epidermidis* were detected in 2 patients (Table 1). The detected bacteria were eliminated after appropriate antibiotic treatment. There were no deaths related to febrile neutropenia during prophylaxis. In addition, The levofloxacin agent adopted in this study was well tolerated. Although elevation of serum transaminase levels was observed in 4 patients, the observed levels were not considered severe. Moreover, no cases of diarrhea associated with *C. difficile* were observed for the duration of the study.

## Discussion

According to previous studies, we assumed that a significant acquisition of levofloxacin-resistant *E. coli* would be present in the fecal flora after prophylaxis in neutropenic patients. However, we did not find a significant acquisition of resistant *E. coli*. Moreover, repeated prophylaxis for more than 2 cycles of chemotherapy did not affect the acquisition of quinolone-resistant *E. coli*. The methods by which quinolone prophylaxis in neutropenic patients possibly affects bacterial antibiotic resistance and patient's prognosis is controversial. Our findings would be suggestive of the benefits and difficulties of quinolone prophylaxis for neutropenia.

In this study, the etiology of bacterial isolates in the fecal flora changed dramatically after quinolone prophylaxis. This etiological change of the fecal flora after the use of quinolone is similar to that of bacteremic isolates in patients who underwent quinolone prophylaxis [17]–[19]; therefore, this suggests that the etiology of bacteremia isolates in patients with febrile neutropenia is mostly attributable to the spices of the fecal flora in these patients.

All *E. coli* isolates detected after the quinolone prophylaxis were resistant to levofloxacin in this study, and most of them were found to have already existed before initiation of prophylaxis. The frequency of levofloxacin-resistant *E. coli* was 16.2% (11/68), of which 7 samples contained ESBL producers (Table 2). ESBL-producing bacteria have been reported resistant to quinolones [20]–[21]. All ESBL-producing *E. coli* isolates detected in this study were also shown to be quinolone-resistant. As previous reports indicate, quinolone-resistant *E. coli* have caused breakthrough bacteremia during prophylaxis with quinolones. Here, it should be stressed that the detection of quinolone-resistant *E. coli* during the prophylaxis is not followed by the increase of mortality at this time [2]–[4]. As suggested by our findings including this data [19], the significant decrease in *E. coli* after quinolone prophylaxis may be related to the lack of increase found in mortality.

Recently, other papers reported interesting findings regarding the field of quinolone prophylaxis for patients with neutropenia [22], [23]. Ng et al. showed that gram-negative isolates, which were all resistant to quinolones, were more frequently recovered from the blood of patients after quinolone prophylaxis than those who had never had prophylaxis. This finding is in contrast with previous observations mentioned above. Ng et al. have implicated that the local prevalence of quinolone-resistant gram-negative bacteria, particularly *E. coli*, may be associated with their results [22]. Thus, a high prevalence of quinolone-resistance among gram-negative bacteria has been suggested to have a strong impact on the selection of those resistant bacteria under quinolone prophylaxis; however, the etiology of the fecal flora was not examined in their study.

This study did not consider the frequency of quinolone-resistant *E. coli* in the fecal flora before initiating prophylaxis. This is a limitation of our study. However, we followed strict inclusion criteria. Patients have a history of antibiotic use within 90 days of first registration for the study were excluded in order to obtain accurate data regarding the etiology of the fecal flora. Yet, our strict criteria created difficulties in recruiting patients for this study. Future studies using larger sample sizes are needed.

In this study, levofloxacin-resistant *E. coli* in the fecal flora were not newly acquired at a significant level after prophylactic administration. Interestingly, nearly 20% of all patients in this study already presented with quinolone-resistant *E. coli* before the initiation of prophylaxis. Based on these findings, the development of bacteremia due to quinolone-resistant *E. coli* in patients with quinolone prophylaxis may be not only caused by the newly acquired quinolone-resistant *E. coli* strains after prophylaxis, but also the resistant strains which already exist before the initiation of prophylaxis. The previous colonization of quinolone-resistant *E. coli* before prophylaxis is likely attributed to the epidemic spread of the resistant strains. Currently, quinolone-resistant *E. coli*, including ESBL producers, have been rapidly spreading worldwide [21], [24]. In our hospital, ∼20% of *E. coli* isolates detected in inpatients and outpatients were ESBL-producers [25]. Therefore, no conclusion can be made as to whether quinolone prophylaxis should be administered for all patients with neutropenia. The high prevalence of quinolone-resistant *E. coli* as a local factor may be a more serious concern for the introduction of the prophylactic use; therefore, continued accumulation of data on both of blood and stool cultures is warranted.
